# SelenzymeRF: updated enzyme suggestion software for unbalanced biochemical reactions

**DOI:** 10.1016/j.csbj.2023.11.039

**Published:** 2023-11-23

**Authors:** Ruth A. Stoney, Erik K.R. Hanko, Pablo Carbonell, Rainer Breitling

**Affiliations:** a Manchester Institute of Biotechnology, Faculty of Science and Engineering, University of Manchester, 131 Princess Street, Manchester M1 7DN, UK; b Institute of Industrial Control Systems and Computing (AI2), Universitat Politècnica de València (UPV), 46022 Valencia, Spain; c Institute for Integrative Systems Biology I2SysBio, Universitat de València-CSIC, 46980 Paterna, Spain

**Keywords:** Retrobiosynthesis, Enzyme selection, Reacting site, Reaction similarity, Unbalanced reactions, RXNMapper

## Abstract

Selenzyme is a retrobiosynthesis tool that suggests candidate enzymes for user query reactions. Enzyme suggestions are based on identical reactions, as well as similar reactions, since enzymes are often capable of promiscuous substrate binding. Selenzyme is a user-friendly, widely used web-tool for ranking enzymes based on reaction similarity and additional features, including the phylogenetic distance between the source species of the enzyme and the intended host. While Selenzyme has proved invaluable in assisting with enzyme selection for known reactions, as well as many novel or orphan reactions, weaknesses have been exposed in its ability to rank functionally related enzymes. Within this update, we introduce a new reaction similarity scoring algorithm, which is used in conjunction with the previous similarity calculation, to improve the accuracy of enzyme suggestions based on non-identical similar reactions, across a range of EC reaction classes. This allows enzymes to be suggested for reactions not found within the database, even if the reaction is unbalanced. A database update was also carried out, to ensure that reaction and enzyme knowledge remains current. This update can be accessed at http://selenzymeRF.synbiochem.co.uk/.

## Highlights

• Updates to the enzyme suggestion webtool Selenzyme improve suggestions for novel or orphan, unbalanced reactions.
• Reacting atoms in unbalanced, novel reactions are located using RXNMapper, an AAM tool designed for unbalanced reactions.
• Reacting site information is integrated into similarity calculations between query reactions and reaction database.
• The median number of shared EC digits between enzymes catalysing test query reactions and suggested enzymes improves from 1 to 3.

## Graphical Abstract

**Figure:**
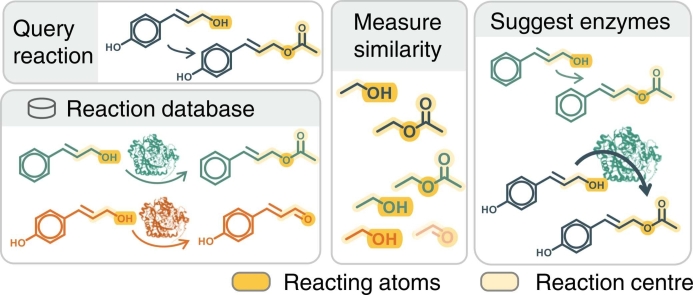

## 1 Introduction

Metabolic engineering involves the generation, engineering and optimization of organisms capable of producing valuable chemicals for pharmacology, food, energy, health and other industries through fermentation processes [1], [2], [3], [4], [5], [6], [7], [8], [9], [10]. The generation of engineered production strains will typically involve the application of synthetic biology tools to pathway design and assembly, followed by the introduction of pathways into industrially convenient hosts. Based on these principles, bioengineers are able to use biomanufacturing pipelines to rapidly design and engineer novel pathways in microbial hosts, using compound-agnostic methods [2].

Retrosynthesis approaches are typically employed to identify the sequences of reactions required to map a target chemical to a convenient precursor compound [11]. These computational processes generate networks of reactions linking the target compound to the desired precursors within the host organism. Pathways are identified by iterating backwards through the network from the target compound to the host metabolite. The output is a set of reactions that would transform the precursors into the target compound.

During retrobiosynthesis the above process is directed towards enzyme-driven reactions; precursor metabolites must either be present within the host metabolome or be suitable for application as a biochemical feedstock [12]. Once the proposed sequence of reactions has been identified, corresponding enzymes must be selected. Enzyme selection is usually either integrated into the retrobiosynthesis algorithm [2], [13], [14], [15] or performed independently, following pathway generation [1], [15], [16].

There are various approaches for generating sets of candidate enzymes for a list of reactions predicted by bioretrosynthesis tools. If the EC class of the enzyme catalysing the reaction is known, this can provide a starting point for selecting suitable enzymes. However, many enzymatic reactions in databases such as KEGG and MetaCyc are not associated with known enzymes. These reactions, which are often found within known biochemical pathways and cause substantial gaps within metabolic networks, are termed “orphan reactions” [17]. Many retrobiosynthetic approaches also generate de novo reactions [2], [11], [13], i.e., reactions that are not yet known to occur in nature (although the search typically is focussed on reaction classes that are found in natural metabolic systems); therefore, a strategy is required to map such orphan and novel reactions to likely candidate enzymes. An approach commonly used to address this issue is the assignment of enzymes to orphan or novel reactions by matching them with similar reactions catalysed by known enzymes [17], [18], assuming that these enzymes could either catalyse the target reaction through substrate promiscuity, or could be engineered to accept the substrate of interest [19].

To search for enzymes capable of performing a novel or orphan reaction, the query reaction is typically compared to reactions present in an enzyme database such as UniProt or Brenda, and the most appropriate reaction–enzyme pair is selected. These approaches typically use fingerprint-based methods capable of encoding the structural and topological properties of substrates and products, or biochemical transformation patterns present within reactions, as the basis for reaction similarity metrics [17].

Selenzyme is one of the popular pieces of software designed to address this need [20]. It is a webtool capable of supplying the user with a ranked list of enzyme suggestions, in response to a reaction rule (such as those from the RetroRules database) or query reaction in SMILES/SMARTS format. Enzyme ranking is based on reaction similarity, along with supplementary metrics including: phylogenetic distance between the intended host organism and the source organisms of the enzyme; UNIPROT protein existence scores indicating the type of evidence supporting the existence of a protein [21]; and the protein evolutionary conservation score indicating cellular importance [20], [22]. For endeavours that include the implementation of novel pathways in several hosts, a Selenzyme update is available to enable phylogenetic distance measures to be taken for multiple host organisms [23].

Selenzyme was designed for the specific purpose of suggesting enzymes for unbalanced reactions, which commonly arise in biochemical conversion reactions when the cofactors for newly proposed reactions are not specified. For example, one source of unbalanced reactions is the output of RetroPath, a state-of-the-art retro(bio)synthesis software [11]. This often happens because currency compounds such as NH_3_, NAD^+^, NADH, Coenzyme A, NAD(P)^+^ and NAD(P)H are excluded from the RetroPath output, as they are not influential for the pathway mapping task. Additionally, unbalanced reactions can arise from data generated from text mining [24], [25], where the leaving group is often omitted from the reaction equation.

In the original version of Selenzyme [20], reaction similarity is calculated based on the full structures of all compounds participating in a reaction, a method that is also adopted by tools such as EC-BLAST and RxnSim [20], [26], [27]. In this approach, similarity between reactions is calculated by applying the Tanimoto method [28] to the fingerprint data generated from each reactions’ chemical components. This process is outlined in depth in Section 2.2.3, but briefly, fingerprints are used to fragment compounds, then the proportion of shared fragments is used to indicate similarity between compound pairs. The reaction similarity is subsequently inferred by calculating a composite similarity score from compound–compound similarity scores. A known complication of these approaches is that large cofactors can adversely affect similarity evaluations. For example, Coenzyme A generates a large number of fragments; therefore, two Coenzyme A molecules bound to different substrate moieties could generate high similarity scores despite participating in very different reactions. Previous methods have provided a user input-based weighting system to circumvent these issues [26] or used full compound comparisons in conjunction with other methods [27].

An alternative approach is to identify the changing bonds within reactions, then focus similarity measures on fingerprint fragments localised around the reacting site. EC-BLAST uses a combined approach, extending similarity measures to the full structures in addition to adopting a more localized approach to the reacting site [27]. In contrast, BridgIT focuses exclusively on the reaction sites, which are located using BNICE.ch-generalized enzyme reaction rules and then represented using BridgIT reaction fingerprints [29]. BridgIT has shown good performance; however, it is limited in its ability to evaluate unbalanced reactions.

While tools that measure similarity around the reacting centre tend to achieve strong performance and avoid the pitfalls generated by large cofactors, they require the reaction to be balanced. This is often an issue, as the cofactors taking part in a reaction may be unknown or are not reported by the upstream retrobiosynthesis software. This issue is circumvented in approaches that use the full structures of all compounds to quantify reaction similarity [20], [26], as these avoid the atom–atom mapping (AAM) that is necessary to identify the reacting site but is difficult in unbalanced reactions. E-zyme2.0 goes some way towards addressing the need to classify unbalanced reactions [30]. It is a tool capable of assigning 3-digit EC numbers to input reactant pairs, stripped of cofactors. While specifying the enzyme class is a helpful start, more specific information is required to select a specific gene, followed by further work identifying the best gene homolog.

Here, we present SelenzymeRF, an update of the Selenzyme software that improves enzyme suggestions through the introduction of the sim_RF algorithm, incorporating RXNMapper [31]. RXNMapper is a machine learning tool designed for AAM in strongly unbalanced reactions. It is uniquely suited for this purpose and has shown superior performance compared to competitor tools: in a recent study comparing RXNMapper to ChemAxon, NameRXN, Indigo, and RDTools AAM performance, RXNMapper emerged as the top performer, generating the most accurate mappings based on a manually mapped evaluation dataset (83.74%) [32].

The impressive accuracy of RXNMapper on independent datasets, made this tool an excellent resource for integration into SelenzymeRF. However, due to inherent obstacles in machine learning approaches, as detailed below, SelenzymeRF was designed to utilize similarity-based measures for downstream reaction classification tasks. The major advantage of similarity-based methods over machine learning approaches is their simplicity and independence from training datasets. The influence of reaction class frequency within the training dataset would obstruct our goal of suggesting optimal enzymes for unusual or unbalanced reactions. Despite advancements in explainable AI, most methods still yield ‘black box’ models that are more challenging to scrutinize. SelenzymeRF combines the benefits of reaction matching accuracy gained from AAM-based methods, while maintaining the ability to assign enzymes to unbalanced reactions, and ensuring that the reaction similarity approaches remain simple enough to be comprehended through visual examination of the reactions.

## 2 Materials and methods

### 2.1 Database preparation

An update of the SelenzymeRF back-end database was completed, incorporating multiple external sources to cover the following data: reaction data including compound SMILES, EC numbers, enzymes and their host organisms; organism taxonomic distances; and enzyme structural data.

The main reaction and chemical information were sourced from MetaNetX [33]. The chemical data is found within the chem_prop file which contains MetaNetX IDs, chemical names, formulas and SMILES, amongst other information. The reaction data is found in the reac_prop file, which contains reaction IDs, chemical equations, the original data source and whether it is a transport reaction (transport reactions were removed).

EC numbers were used to link reactions to enzymes. The enzyme data used within this study came from two sources: Brenda [34] and EXpasy [35]. Datasets were downloaded and all EC–enzyme–organism connections were extracted. Reactions containing these EC numbers were annotated with the corresponding enzymes in the SelenzymeRF database, within the seq_org.csv file. Further information regarding the protein and host organism was retrieved from UniProt [21]. Finally, phylogenetic distances between organisms were extracted from the NCBI database [36]. The workflow for the update, along with full instructions is available at https://github.com/synbiochem/selenzyme.

### 2.2 Reaction processing

**Fig. 1:**
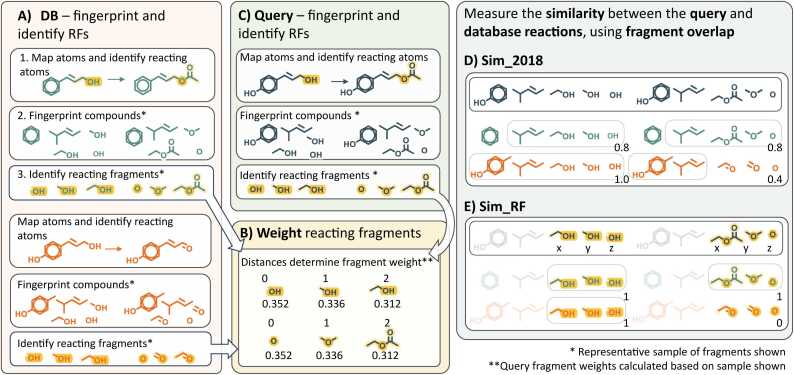
Measuring reaction similarity using compound fragments. A) All reactions within the SelenzymeRF database (DB) were processed in the following ways, prior to software release: RXNMapper performed AAM between reaction substrates and products; Morgan fingerprinting of DB compounds was performed; RFs were identified as fragments containing reacting atoms. B) Size dependent weights were calculated for RFs. C) In response to a user input query the processing steps listed in A and B are applied to the query reaction. D) The most similar compounds within the query reaction and each DB reaction are aligned using sim_2018, then the compound similarity is calculated as the proportion of shared fragments between each compound pair. E) The similarity of the reacting fragments is calculated by sim_RF, based on shared RFs between the query and DB compounds.

To identify suitable enzymes for user query reactions, similar reactions must be identified from within the database. In both Selenzyme and SelenzymeRF, the calculation of reaction similarity involves processing both the substrates and products into chemical fingerprint fragments (Fig. 1. A2). These fingerprint fragments represent overlapping chemical substructures. The details of fingerprint generation are outlined in section 2.2.1.

A significant enhancement introduced in SelenzymeRF is the identification of reacting atoms, enabling the classification and weighting of fragments based on their proximity to the reacting site. AAM is performed using RXNMapper (see Section 2.2.2), then RDKit is employed to detect reacting atoms within each compound (Fig. 1. A1). This information is then used to classify the fingerprint fragments containing one or more reacting atoms as reacting fragments (RF), as illustrated in Fig. 1. A3.

These RFs will vary in size, with some being small and highly localised to the reacting atoms, while others are larger and encompass more of the surrounding chemical substructures. The reacting atoms may also appear at any position within the RF; therefore, some RFs will have central reacting atoms, while others will contain reacting atoms on the periphery of the fragment. A weighting system was introduced to favour fragments that provide highly localised representations of the reacting atoms, based on the distance between the reacting atom and the most distant non-reacting atom in the fragment (Fig. 1. B, further details in Section 2.2.3).

This process of reducing reactions into sets of substrate and product fragments, and then classifying each fragment based on the localisation of any reacting atoms, was precomputed for all the reactions in the database (Fig. 1. A). These steps are also followed when the user submits a query reaction (Fig. 1. C).

#### 2.2.1 Fingerprint generation

Morgan fingerprints are generated from MetaNetX SMILES data, using the GetMorganFingerprint function from the rdkit.AllChem package, with the maximum fragment radius set to 8 and includeRingMembership set to False (to allow for linear to ring transformations). The fingerprint data for the database was stored as npz files.

Compared to the original version of Selenzyme, the option to allow the user a choice of fingerprinting method was removed, with only Morgan fingerprints supplied. This change was implemented to make the tool more user friendly without reducing performance, as Morgan fingerprinting has been shown to outperform other fingerprinting methods [37], [38]. In addition, updating the database and supplying RFs increased the data storage requirements, and removing the choice of fingerprint methods helped compensate for these changes.

#### 2.2.2 Atom–atom mapping using RXNMapper

For each reaction it is necessary to discover the precise location within each chemical component that changed during the reaction. The update employs RXNMapper, a reaction-mapper tool that uses self-supervised neural networks to identify corresponding atoms between substrates and products, even in strongly unbalanced reactions [31]. This package is unable to accommodate SMILES containing the star regular expression wildcard; therefore, compounds that included a ‘* ’ within their SMILES string were excluded from AAM. Reactions with no applicable SMILES strings for the substrates or products are still searchable using sim_2018. The directionality of the RXNMapper input SMILES string was also arranged to ensure that the unbalanced reactions had a higher atom count on the left of the equation, in compliance with Schwaller et al. (2021). We then identified the reacting atoms using the Rdkit AllChem package along with the mapped SMILES.

#### 2.2.3 RF generation and weighting

As detailed in the original publication, the sim_2018 score is based upon the Tanimoto similarity of compound fragments. This algorithm pairs compounds in the query reaction to compounds in the database reaction (Fig. 1.D.) As illustrated in Fig. 1. B a weighting system was implemented to score RFs generated from the query compound. For each pair of query and database compounds, RFs from the query compound that were also present in the database compound were identified, and the total sum of their weights calculated (Fig. 1. E).

The weighting system was designed to increase the influence of small RFs that are highly specific to the reacting site and reduce the influence of larger less specific RFs (Eqs. (1) & (2), Fig. 1. E). To measure RF specificity, we calculated the maximum distance from a reacting atom to the furthest atom within the compound. A reverse sigmoid function was employed to assign weights, with the point of inflection (*i*) set to 5 and the slope (*k*) set to 0.5 (Eq. (1)). These parameters were determined to optimize the fraction of non-identical enzyme suggestions matching 3 or more EC digits (described in section 2.5). By setting the point of inflection to five we ensured that fragments where all the atoms were less than five bonds from the reacting atom received relatively high scores, while larger fragments received substantially lower scores.

Finally, to account for the number of RFs and maximum RF size being dependent on the compound size and structure, linear sum normalization was used to ensure that all the weights in each compound had a total sum of 1 (Eq. (3)) [39]. This process involves dividing all the weights by their sum.

(1) $$f\left(x\right)=\frac{1}{1+e^{k(x-i)}}$$

Eq. (1) Reverse Sigmoid Function, where x is the maximum distance between the reacting atom(s) and all other atoms, k is the slope (0.5) and i is the point of inflection (5).

(2) $$s=\left[\begin{array}{c} f\left(x_{1}\right)\\ f\left(x_{2}\right)\\ ...\\ f\left(x_{n-1}\right)\\ f\left(x_{n}\right) \end{array}\right]$$

Eq. (2) Scores (s) are generated based on the reverse log of the distance between the reacting atom and the furthest non-reacting atom, using function f.

(3) $$s_{\mathrm{norm}}=\left[\begin{array}{c} s_{1}/\sum s\\ s_{2}/\sum s\\ ...\\ s_{n-1}/\sum s\\ \;s_{n}/\sum s \end{array}\right]$$

Eq. (3) Scores are normalised (s_norm_) to ensure that they have a sum of 1.

### 2.3 Reaction similarity calculations

#### 2.3.1 sim_2018

The sim_2018 algorithm is used to calculate reaction similarities based on compound similarities; this is unchanged from the original publication and is used as a point of reference when assessing the performance of the newly developed algorithm described below. Tanimoto distance matrices were generated between the compounds in the query reaction and the compounds in the database. The Tanimoto similarity was used to calculate the proportion of fingerprint fragments that are shared between two compounds [40]. The similarity between each database reaction and the query reaction was calculated using a greedy heuristic to match each compound in the query to the closest compound within the database reaction. The Tanimoto scores for these best-match compound pairings were compiled into similarity scores for the substrates and products, then averaged to give a single sim_2018 score (see the original Selenzyme publication [20]). The best-match compound pairings between the query compounds and the database compounds are also reused by the sim_RF algorithm.

#### 2.3.2 sim_RF

The best-match compound pairings generated by sim_2018 are reused by sim_RF during the calculation of the reacting fragment similarity (Eq. (4)). The similarity is calculated as the proportion of query compound RFs present in the database compound RFs, weighted by the fragment radius (see Section 2.2.3). An asymmetric similarity measure was selected, rather than the Tanimoto measure, because unbalanced query reactions may be missing some reacting atoms present in the database reaction. This is due to the inherent limitation that only atoms present in the substrate and product can be mapped; therefore, in unbalanced reactions some atoms are likely to be unmapped. This may result in compounds containing sections in which the atoms are mapped and sections in which the atoms are unmapped. If the unmapped atoms are involved in additional transformations with unseen compounds, these will not be represented in the RFs. This can be explained using the fictional database reaction A + B + C → ABC; if the incomplete query reaction B → ABC was used as a query reaction, the reacting atoms representing changes between A and B would be found, but reacting atoms connecting B and C would be lost.

The mean RF similarity is calculated for the substrates and products of the query reaction. Finally, the square root of the product is calculated for the substrate and product scores, to generate an overall sim_RF score for the query and database reactions (Eq. (4)). This measure penalises substrate-RF-score/product-RF-score pairs in which one score is high and the other is low. For ease of use, columns containing sim_2018 and sim_RF scores have been added to the results output table presented to the user.

(4) $$\mathrm{si}m_{\mathrm{RF}}=\sqrt{\left(\frac{\sum S}{N}*\frac{\sum P}{N}\right)}$$

Eq. (4) The sim_RF score is generated by calculating the mean score for the substrates (S) and the products (P), multiplying these two figures and finally applying the square root.

### 2.4 Accuracy testing of sim_RF

To test the ability of sim_2018 and sim_RF to suggest suitable non-identical reactions, KEGG pathway modules were used to generate sets of unbalanced reactions. KEGG modules provide sets of reactions comprising a particular pathway with co-factors excluded. A set of 12 modules were selected, covering core metabolism and a range of illustrative biosynthetic pathways. To challenge the algorithm’s ability to handle unbalanced reactions, any reactions where multiple substrates or products were utilized by the pathway, were split into multiple query SMILES. It was necessary to exclude 5 reactions from analysis, because the SMILES of their chemical components contained ‘* ’, making then impossible to process using RXNmapper. This process generated a set of 101 reaction SMILES from the following KEGG Pathway modules: M00001 – Glycolysis (10 reactions), M00004 – Pentose phosphate pathway (18 reactions), M00009 – Citrate cycle (9 reactions), M00018 – Threonine biosynthesis (5 reactions), M00022 – Shikimate pathway (8 reactions), M00101 – Cholesterol biosynthesis (13 reactions), M00110 – C19/C18-Steroid hormone biosynthesis (6 reactions), M00125 – Riboflavin biosynthesis (8 reactions), M00137 – Flavanone biosynthesis (5 reactions), M00138 – Flavonoid biosynthesis (3 reactions), M00372 – Abscisic acid biosynthesis (8 reactions), and M00944 – Morphine biosynthesis (9 reactions). One reaction was present in both glycolysis and the pentose phosphate pathway modules.

These SMILES were used to query the SelenzymeRF REST API, using the ‘targets’ parameter to return 5000 results per query. The closest non-identical reactions based on the sim_RF and sim_2018 algorithms were selected from the output data. Reactions that had a sim_2018 score greater than 0.99 were considered identical and were used as reference for the correct EC numbers. Identical reactions were excluded from the accuracy assessment and the most similar non-identical reactions were selected. The most similar reactions were defined as the reaction(s) with the highest sim_2018 or sim_RF score (rounded to 4 decimal places), after exact matches were removed. If multiple EC numbers were available for a query reaction or if multiple reactions with identical scores were selected, the highest scoring EC comparison was recorded.

### 2.5 Parameter optimization

To determine the optimal parameters for the reverse sigmoid function’s slope and point of inflection, a range of parameters was tested. Accuracy was assessed using the method described in section 2.4 modified to include only the top 50 reactions. The following slope parameters were tested: 0.5, 1 and 2; along with the following set of inflection point parameters: 0, 1, 3 and 5. The parameters that yielded the highest percentage of non-identical enzyme suggestions matching 3 or 4 EC digits were selected (Table 1).

**Table 1:** Effect of reverse logistic parameters on sim_RF accuracy. Table shows the percentage of reactions where the top scoring non-identical RF matched 3 or 4 EC digits.

	Inflection (*i*)					
Slope (*k*)		0	1	3	5	total
	0.5	61.2	63.3	61.2	**63.3**	**249**
	1	59.2	59.2	61.3	63.3	243
	2	55.1	59.2	59.2	63.3	236.8
	total	175.5	181.7	181.7	189.9	

## 3 Results and discussion

### 3.1 Database updates

**Fig. 2:**
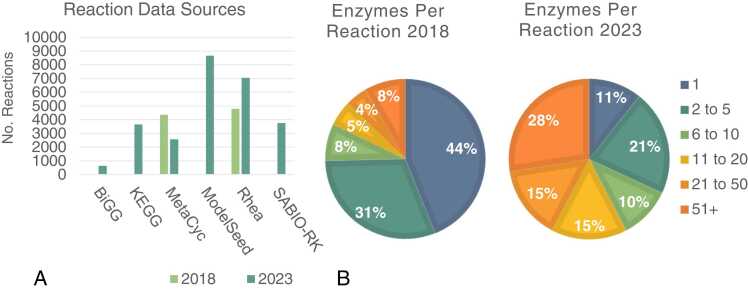
A) Distribution of reactions extracted from external databases in the 2018 and 2023 datasets. B) Distribution of enzyme annotations per reaction within the 2018 and 2023 datasets. Following the update, the percentage of reactions annotated with a single enzyme is reduced from 44% to 11%, with overall reductions seen in the percentage of reactions annotated with fewer than 6 enzymes.

The following improvements to the database are a result of the data update: the total number of reactions was increased from 9141 to 26,342 (188%); the number of enzymes was increased from 191,361 to 245,500 (28%); the number of compounds increased from 8090 to 15,496 (92%); and the number of taxonomic species increased from 6538 to 8305 (27%). Redundancy within the reaction datasets was low, with the update containing 23,840 unique reactions (90.5%), compared with 8409 (92.0%) in the original dataset. The distribution of reactions from various databases is shown in Fig. 2A; this update includes reactions from BiGG [41], KEGG [42], MetaCyc [43], ModelSeed [44], Rhea [45] and SABIO-RK [46], while the original version sourced reactions from KEGG, MetaCyc and Rhea. An important feature of SelenzymeRF is its ability to rank gene homologs, based on various metrics, some of which were described in the introduction. The median number of enzymes per reaction was increased from 2 to 16 in the 2023 dataset (Fig. 2B). Following the update, a reduction can be seen in the percentage of reactions annotated with fewer than 6 enzymes. Fig. 2A illustrates that the number of MetaCyc reactions present in the updated dataset is lower compared to the original dataset. This difference is primarily due to the presence of non-standard EC numbers in the original dataset, which were either incomplete or contained letters, impeding enzyme retrieval in the update. Nevertheless, the updated dataset still encompasses almost twice as many reactions as the original dataset when considering all data sources.

### 3.2 Improved accuracy of reaction similarity algorithms

**Fig. 3:**
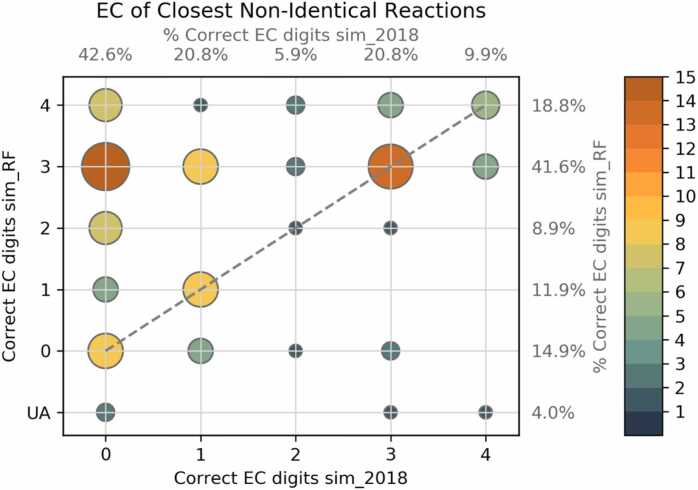
The number of correct EC numbers was matched for 101 non-identical best matching reactions, using the sim_2018 and the sim_RF algorithms. The annotations on the top and right axes indicate the marginal probabilities of the closest non-identical reaction being correct to 1–4 EC digits (for sim_2018 and sim_RF, respectively). Within the y axis, UA indicates that reacting fragments were unavailable. A clear improvement in the probability of identifying enzymes with two or more matching EC digits can be seen for the new sim_RF algorithm, increasing from 36.6% to 69.3%. The probability of the closest non-identical reaction sharing zero EC digits with the query reaction is reduced from 42.6% to 14.9%. For 4.0% of query reactions, RXNMapper was unable to perform atom-atom mapping (AAM).

The sim_RF and sim_2018 algorithms were tested using 101 unbalanced KEGG reactions. While both algorithms were able to consistently find exact matches for the queried reactions, this test aimed to assess the accuracy of sim_RF and sim_2018 to accurately select similar non-identical reactions. Accuracy was measured using the number of shared EC digits between the enzymes catalysing the query reaction and the closest non-identical reactions. Sim_RF demonstrates a 69.3% probability of matching 2 or more EC digits in the most similar non-identical reaction (Fig. 3), marking a substantial 89% improvement over sim_2018 (36.6%). The median number of accurately predicted EC digits is 3 for the sim_RF, while for sim_2018 it stands at 1. In 11.9% of instances, sim_2018 outperforms sim_RF, and in 4% of tests, generation of RFs was unsuccessful. To minimize the probability of overlooking plausible candidates in these rare cases, we recommend considering both similarity ranking methods when selecting enzyme candidates.

Examination of instances in which sim_RF performed worse than sim_2018 revealed that 5 of the 12 reactions were incorrectly mapped to an isomerase reaction by the sim_RF algorithm. Of these 5 reactions, 4 query reactions featured polycyclic compounds. Isomeric reactions between linear and cyclic isoforms generate high numbers of reacting atoms, making them particularly vulnerable to RF errors. Full details of these results are available in Tables S1 and S2.

### 3.3 Case studies

**Fig. 4:**
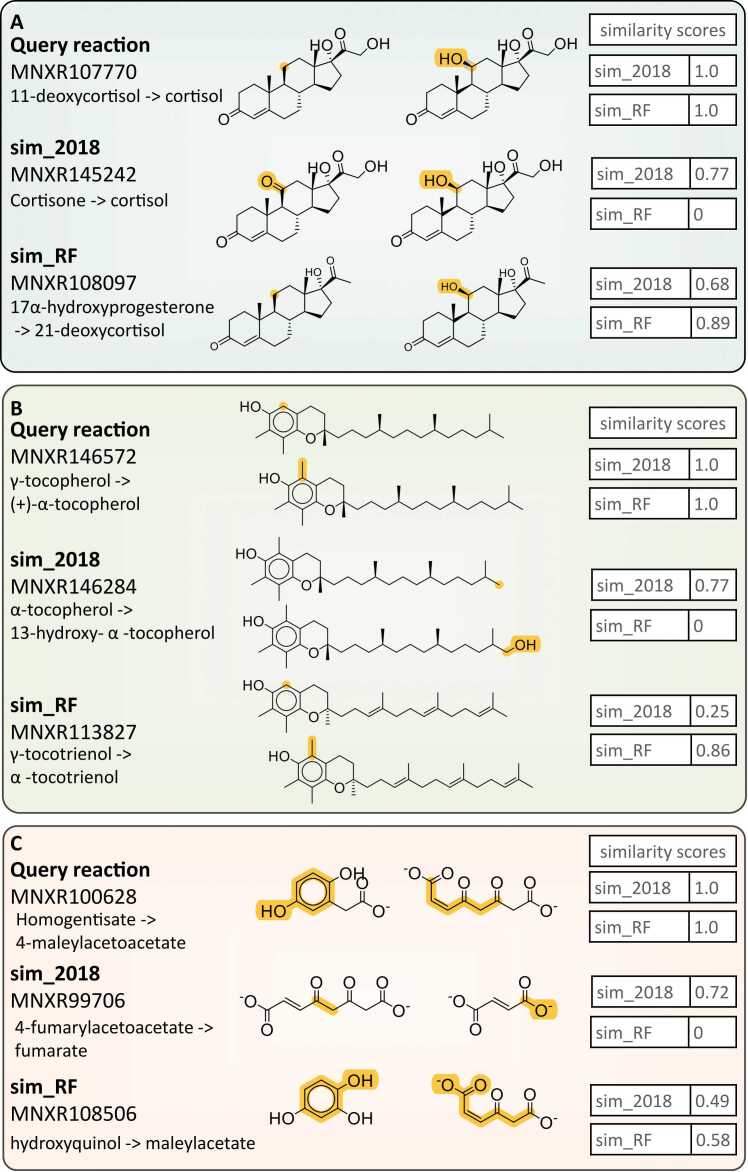
Comparison between query reactions and the most similar non-identical reactions identified by sim_2018 and sim_RF. The reacting centres are highlighted within each reaction. The MetaNetX identifiers, as well as the similarity scores generated by sim_2018 and sim_RF are given for each reaction. A) The query reaction of 11-deoxycortisol to cortisol. The closest non-identical reaction generated by sim_RF was 17α-hydroxyprogesterone to 21-deoxycortisol, which is more closely related to the query reaction than the reaction returned by sim_2018 (cortisone to cortisol). B) The query reaction of γ-tocopherol to (+)-α-tocopherol. The closest non-identical reaction generated by sim_RF was γ-tocotrienol to α-tocotrienol, which is more closely related to the query reaction than the reaction returned by sim_2018 (13-α-tocopherol to hydroxy-α-tocopherol). C) The query reaction of homogentisate to 4-maleylacetoacetate. The closest non-identical reaction generated by sim_RF was hydroxyquinol to maleylacetate, which is more closely related to the query reaction than the reaction returned by sim_2018 (4-fumarylacetoacetate to fumarate).

The ability of sim_2018 and sim_RF to rank reactions based on perceived similarity is illustrated in Fig. 4, using three examples of query reactions. The query reaction at the top of each example shows the compounds used to create the query SMILES. As expected, the reactions used to generate the query SMILES received a similarity score of 1 for all ranking metrics. The two reactions depicted below each query reaction are the highest scoring, non-identical reactions according to sim_2018 and sim_RF.

For the first example, we tested the transformation of 11-deoxycortisol to cortisol (Fig. 4A). Sim_2018 selected the transformation of cortisone to cortisol as the most similar, non-identical reaction, based on the overall similarity of the substrates and products. The sim_2018 score is high (0.77) because both reactions generate the same product, and the substrates (cortisone and 11-deoxycortisol) only differ by a single keto group. However, the reaction suggested by sim_2018 is a reduction rather than a hydroxylation, making the suggested enzyme unlikely to catalyse the reaction of interest. The novel sim_RF approach avoids these misleading suggestions: here, the dissimilarity between the chemical transformations is reflected in the lower sim_RF score of 0. This score reflects the fact that, while the reacting fragments from the product match, those from the substrates do not, therefore, the multiplication of these two scores gives an overall score of zero.

In contrast, the most similar non-identical reaction identified by sim_RF is the hydroxylation of 17α-hydroxyprogesterone to 21-deoxycortisol. In this reaction, both the substrate and product differ from the query reaction, reducing the sim_2018 score to 0.68. However, the hydroxy group that distinguishes 17α-hydroxyprogesterone from 11-dehydroxycortisol is located far from the reacting site, allowing the sim_RF score to remain high. It is apparent that the reaction selected by sim_RF is more appropriate than the reaction returned by sim_2018. This is confirmed by examination of the EC numbers; the reaction returned by sim_RF has the same EC number as the query reaction (1.14.15.4), while the reaction returned by sim_2018 does not (1.1.1.146).

For the second example, we investigated the transformation of vitamin E from the gamma form most commonly consumed in a plant-based diet (γ-tocopherol) to the predominant alpha form found in human and animal tissues (α-tocopherol, Fig. 4B, [47]). The most similar, non-identical reaction returned by sim_2018 is the transformation of α-tocopherol to 13-hydroxy-α-tocopherol. This reaction encompasses the hydroxylation of the α-tocopherol side chain, at the opposite end of the compound to the query reaction centre. Again, the new SelenzymeRF approach avoids this misleading suggestion: reflecting the dissimilarity of the sim_2018 reaction to the query reaction, the sim_RF score is 0. The most similar, non-identical reaction generated by sim_RF applies the correct reaction to the substrate γ-tocotrienol. This substrate is distinguished from γ-tocopherol by its unsaturated side chain; however, these differences are distal from the query reacting site, and it is plausible to expect that the enzyme would promiscuously accept γ-tocopherol as an alternative substrate or could be engineered to do so.

The final example illustrates the transformation of homogentisate to 4-maleylacetoacetate (Fig. 4C), a reaction present in the tyrosine degradation pathway. The reaction selected as the most similar non-identical reaction by sim_2018 concerns 4-fumarylacetoacetate, which is similar to 4-maleylacetoacetate, involved in an unrelated transformation. The reaction returned by sim_RF is the dioxygenation of hydroxyquinol, a more plausible candidate reaction for the query reaction.

**Fig. 5:**
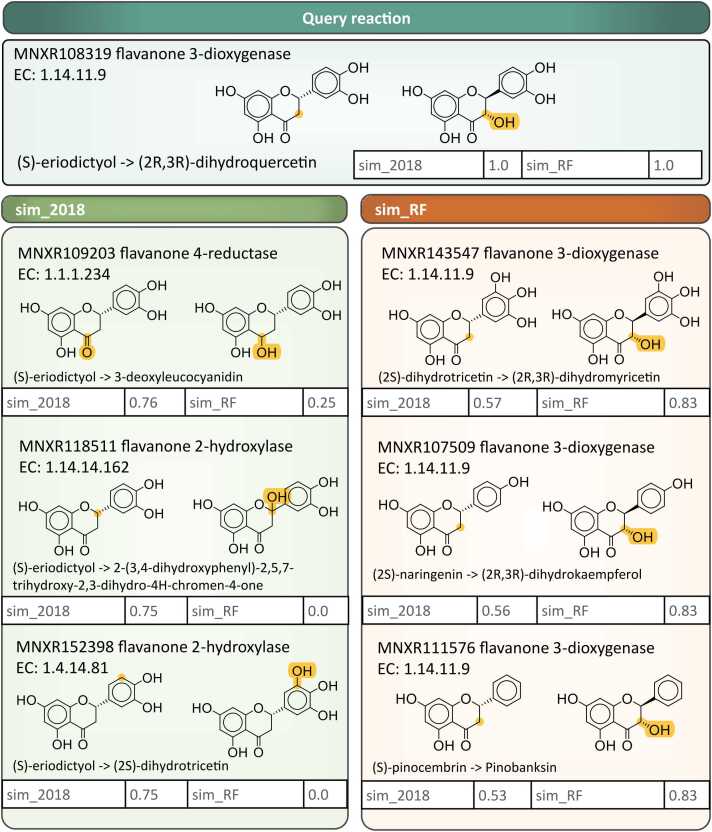
Examination of the enzymes and EC numbers associated with sim_2018 and sim_RF top suggestions.

This ability of sim_RF to generate better enzyme candidates is illustrated by the three most similar non-identical reactions identified by sim_2018 and sim_RF for the transformation of (S)-eriodictyol to (2 R,3 R)-dihydroquercetin (Fig. 5). All three reactions suggested by sim_RF are catalysed by the same flavanone 3-dioxygenase enzymes as the query (A0A4D6Q9B0 and A0A4D6Q4T7). In contrast, the closest non-identical reactions from sim_2018 are associated with other enzymes, such as flavanone 4-reductase and flavanone 2-hydrolase homologs, which introduce hydroxyl groups at positions that are different to the ones in the query reactions.

This tendency for similar non-identical reactions from sim_RF to be associated with the same enzymes as the exact match, while the similar non-identical sim_2018 reactions correspond to different enzymes is also seen in the previous examples. In the examples shown in Fig. 4A and Fig. 4B, the reactions returned by sim_RF are associated with the same enzymes as the query reaction, while the reactions from sim_2018 are linked to different sets of enzymes. The last example shows an interesting case (Fig. 4C), in which the enzyme corresponding to the query (Homogentisate 1,2-dioxygenase) is specific to the query reaction (EC number 1.13.11.5). In this instance, the software must detect reactions with alternative enzymes. This example shows that even in the absence of alternative reactions for a given enzyme, sim_RF, the algorithm implemented in the updated SelenzymeRF, often suggests more plausible enzyme options than its predecessor sim_2018.

## 4 Conclusion

The major improvement between Selenzyme and SelenzymeRF is the consideration of the reacting centre during the calculation of reaction similarity. The original version of Selenzyme calculated similarity based solely on the overall similarity of the reacting compounds, which could cause issues distinguishing between dissimilar transformations of similar compounds. By identifying the location of the reacting site using RXNMapper and applying weights to the area surrounding the reacting centre, SelenzymeRF is able to focus on the chemical transformation occurring within the query reaction, improving the software’s ability to suggest enzyme candidates based on similar non-identical reactions. The release of SelenzymeRF includes a database update that has increased the number of available reactions by 188%. Additionally, the development of core algorithms has resulted in substantially increased accuracy in the enzyme similarity rankings.

SelenzymeRF can be effectively integrated with other complementary software. EC prediction tools such as E-ZYME [30] or Theia [48] can be used to classify the query reaction, and the obtained prediction can then be utilized to refine the SelenzymeRF output. AutoDocker [49] and related software could also be used downstream of SelenzymeRF to assess the binding suitability between the reaction compounds and the suggested enzymes, e.g., by identifying cases where the size of a substrate is clearly too large to fit into the binding pocket of a candidate enzyme. These pieces of software provide additional information that supplements the enzyme proposals of SelenzymeRF. Furthermore, promising studies predicting site-of-metabolism likelihood for AAM reactions [50] present intriguing possibilities for development, building upon the implemented reaction mapping technology.

## Funding Source Declaration

This project has received funding from the European Union’s Horizon 2020 research and innovation programme under grant agreement 814408 SHIKIFACTORY100—Modular cell factories for the production of 100 compounds from the shikimate pathway.

We acknowledge funding from the Biotechnology and Biological Sciences Research Council (10.13039/501100000268BBSRC) under grant BB/M017702/1 “Centre for synthetic biology of fine and speciality chemicals (SYNBIOCHEM)”.

Pablo Carbonell was supported by the Spanish Ministry of Universities (UNI/551/2021), grant number UP2021-036 funded by European Union-Next generation EU.

Pablo Carbonell acknowledges funding from Generalitat Valenciana through grant CIAICO/2021/159 (SmartBioFab).

Pablo Carbonell acknowledges MCIN/AEI/10.13039/501100011033 and NextGenerationEU/ PRTR funding through grant TED2021-131049B-I00 (BioEcoDBTL).

## CRediT authorship contribution statement

**Ruth A. Stoney**: Methodology, Software, Formal analysis, Investigation, Resources, Data curation, Writing − original draft, Writing − review & editing, Visualization. **Erik K.R. Hanko**: Validation, Writing − review & editing, Visualization. **Pablo Carbonell**: Conceptualization, Methodology, Software, Resources, Data curation, Writing − review & editing, Funding acquisition. **Rainer Breitling**: Conceptualization, Methodology, Validation, Resources, Writing − review & editing, Supervision, Project administration, Funding acquisition.

## Declaration of Competing Interest

The authors declare that they have no known competing financial interests or personal relationships that could have appeared to influence the work reported in this paper.

## References

[1] Otero-Muras I., Carbonell P. (2021). Automated engineering of synthetic metabolic pathways for efficient biomanufacturing. Metab Eng.

[2] Robinson C.J. (2020). Rapid prototyping of microbial production strains for the biomanufacture of potential materials monomers. Metab Eng.

[3] Del Carratore F., Hanko E.K., Breitling R., Takano E. (2022). Biotechnological application of Streptomyces for the production of clinical drugs and other bioactive molecules. Curr Opin Biotechnol.

[4] Chauhan S. (2023). Engineered Microbial Systems for the Production of Fuels and Industrially Important Chemicals. Sarangi PK Editor Biorefinery Prod Fuels Platf Chem.

[5] Ko Y.S. (2020). Tools and strategies of systems metabolic engineering for the development of microbial cell factories for chemical production. Chem Soc Rev.

[6] Becker J., Wittmann C. (2020). Microbial production of extremolytes - high-value active ingredients for nutrition, health care, and well-being. Curr Opin Biotechnol.

[7] Babaei M. (2020). Metabolic engineering of Saccharomyces cerevisiae for rosmarinic acid production. ACS Synth Biol.

[8] Milne N. (2020). Metabolic engineering of Saccharomyces cerevisiae for the de novo production of psilocybin and related tryptamine derivatives. Metab Eng.

[9] Sáez-Sáez J. (2020). Engineering the oleaginous yeast Yarrowia lipolytica for high-level resveratrol production. Metab Eng.

[10] Dunstan M.S. (2020). Engineering Escherichia coli towards de novo production of gatekeeper (2S)-flavanones: naringenin, pinocembrin, eriodictyol and homoeriodictyol. Synth Biol.

[11] Delépine B., Duigou T., Carbonell P., Faulon J.L. (2018). RetroPath2. 0: A retrosynthesis workflow for metabolic engineers. Metab Eng.

[12] Hadadi N., Hatzimanikatis V. (2015). Design of computational retrobiosynthesis tools for the design of de novo synthetic pathways. Curr Opin Chem Biol.

[13] Hafner J., MohammadiPeyhani H., Sveshnikova A., Scheidegger A., Hatzimanikatis V. (2020). Updated ATLAS of biochemistry with new metabolites and improved enzyme prediction power. ACS Synth Biol.

[14] Jeffryes J.G. (2015). MINEs: open access databases of computationally predicted enzyme promiscuity products for untargeted metabolomics. J Chemin-.

[15] Kuwahara H., Alazmi M., Cui X., Gao X. (2016). MRE: a web tool to suggest foreign enzymes for the biosynthesis pathway design with competing endogenous reactions in mind. Nucleic Acids Res.

[16] Ding S. (2020). novoPathFinder: a webserver of designing novel-pathway with integrating GEM-model. Nucleic Acids Res.

[17] Zhang Z., Li C. (2022). Enzyme annotation for orphan reactions and its applications in biomanufacturing. Green Chem Eng.

[18] Moriya Y. (2010). PathPred: an enzyme-catalyzed metabolic pathway prediction server. Nucleic Acids Res.

[19] Yu T. (2023). Machine learning-enabled retrobiosynthesis of molecules. Nat Catal.

[20] Carbonell P. (2018). Selenzyme: enzyme selection tool for pathway design. Bioinformatics.

[21] (2023) UniProt: the Universal Protein knowledgebase in 2023. Nucleic Acids Research 51: D523-D531. 10.1093/nar/gkac1052 PMC9825514 36408920

[22] Landau M. (2005). ConSurf 2005: the projection of evolutionary conservation scores of residues on protein structures. Nucleic Acids Res.

[23] Camarena M., and Carbonell P. (2021) Developing an enzyme selection tool supporting multiple hosts contexts. bioRxiv.

[24] Lowe D.M. (2012). Extr Chem Struct React Lit.

[25] Bort W. (2021). Discovery of novel chemical reactions by deep generative recurrent neural network. Sci Rep.

[26] Giri V., Sivakumar T.V., Cho K.M., Kim T.Y., Bhaduri A. (2015). RxnSim: a tool to compare biochemical reactions. Bioinformatics.

[27] Rahman S.A., Cuesta S.M., Furnham N., Holliday G.L., Thornton J.M. (2014). EC-BLAST: a tool to automatically search and compare enzyme reactions. Nat Methods.

[28] Willett P. (2005). Searching techniques for databases of two-and three-dimensional chemical structures. J Med Chem.

[29] Hadadi N., MohammadiPeyhani H., Miskovic L., Seijo M., Hatzimanikatis V. (2019). Enzyme annotation for orphan and novel reactions using knowledge of substrate reactive sites. Proc Natl Acad Sci.

[30] Yamanishi Y., Hattori M., Kotera M., Goto S., Kanehisa M. (2009). E-zyme: predicting potential EC numbers from the chemical transformation pattern of substrate-product pairs. Bioinformatics.

[31] Schwaller P., Hoover B., Reymond J.L., Strobelt H., Laino T. (2021). Extraction of organic chemistry grammar from unsupervised learning of chemical reactions. Sci Adv.

[32] Lin A. (2022). Atom‐to‐atom mapping: a benchmarking study of popular mapping algorithms and consensus strategies. Mol Inform.

[33] Moretti S., Tran V.D.T., Mehl F., Ibberson M., Pagni M. (2021). MetaNetX/MNXref: unified namespace for metabolites and biochemical reactions in the context of metabolic models. Nucleic Acids Res.

[34] Chang A. (2021). BRENDA, the ELIXIR core data resource in 2021: new developments and updates. Nucleic Acids Res.

[35] Bairoch A. (2000). The ENZYME database in 2000. Nucleic Acids Res.

[36] Federhen S., Bethesda M.D. (2003). National Center for Biotechnology Information.

[37] Capecchi A., Probst D., Reymond J.L. (2020). One molecular fingerprint to rule them all: drugs, biomolecules, and the metabolome. J Chemin-.

[38] Schneider N., Fechner N., Landrum G.A., Stiefl N. (2017). Chemical topic modeling: Exploring molecular data sets using a common text-mining approach. J Chem Inf Model.

[39] Mhlanga S.T., Lall M. (2022). Influence of Normalization Techniques on Multi-criteria Decision-making Methods. J Phys: Conf Ser.

[40] Bajusz D., Rácz A., Héberger K. (2015). Why is Tanimoto index an appropriate choice for fingerprint-based similarity calculations?. J Chemin-.

[41] Norsigian C.J. (2020). BiGG Models 2020: multi-strain genome-scale models and expansion across the phylogenetic tree. Nucleic Acids Res.

[42] Kanehisa M., Goto S. (2000). KEGG: kyoto encyclopedia of genes and genomes. Nucleic Acids Res.

[43] Caspi R. (2020). The MetaCyc database of metabolic pathways and enzymes-a 2019 update. Nucleic Acids Res.

[44] Henry C.S. (2010). High-throughput generation, optimization and analysis of genome-scale metabolic models. Nat Biotechnol.

[45] Bansal P. (2022). Rhea, the reaction knowledgebase in 2022. Nucleic Acids Res.

[46] Wittig U., Rey M., Weidemann A., Kania R., Müller W. (2018). SABIO-RK: an updated resource for manually curated biochemical reaction kinetics. Nucleic Acids Res.

[47] Jiang Q., Christen S., Shigenaga M.K., Ames B.N. (2001). γ-Tocopherol, the major form of vitamin E in the US diet, deserves more attention. Am J Clin Nutr.

[48] Probst D. (2023) Explainable prediction of catalysing enzymes from reactions using multilayer perceptrons. bioRxiv.

[49] Morris G.M. (2009). AutoDock4 and AutoDockTools4: Automated docking with selective receptor flexibility. J Comput Chem.

[50] Porokhin V., Liu L.-P., Hassoun S. (2023). Using graph neural networks for site-of-metabolism prediction and its applications to ranking promiscuous enzymatic products. Bioinformatics.

