# Supplementary material for: SelenzymeRF: updated enzyme suggestion software for unbalanced biochemical reactions
Source: Comput Struct Biotechnol J. 2023 Nov 23;21:5868–76. doi: 10.1016/j.csbj.2023.11.039 (PMC10697999; doi:10.1016/j.csbj.2023.11.039)
Supplement: Supplementary file 1 — Supplementary material [file mmc1.pdf]

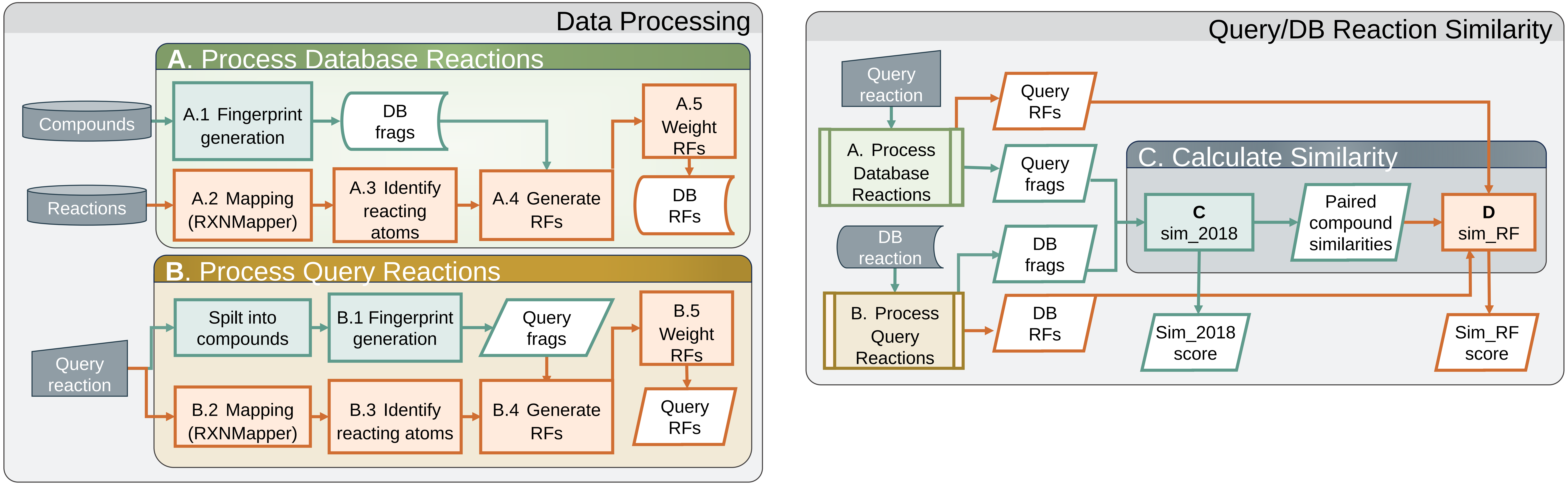
Data Processing
A. Process Database Reactions
Compounds
A.1 Fingerprint generation
DB frags
A.5 Weight RFs
Reactions
A.2 Mapping (RXNMapper)
A.3 Identify reacting atoms
A.4 Generate RFs
DB RFs
B. Process Query Reactions
Spilt into compounds
B.1 Fingerprint generation
Query frags
B.5 Weight RFs
Query reaction
B.2 Mapping (RXNMapper)
B.3 Identify reacting atoms
B.4 Generate RFs
Query RFs
Query/DB Reaction Similarity
Query reaction
Query RFs
A. Process Database Reactions
Query frags
C. Calculate Similarity
DB reaction
DB frags
C sim_2018
Paired compound similarities
D sim_RF
B. Process Query Reactions
DB RFs
Sim_2018 score
Sim_RF score
