# Supplementary material for: SelenzymeRF: updated enzyme suggestion software for unbalanced biochemical reactions
Source: Comput Struct Biotechnol J. 2023 Nov 23;21:5868–76. doi: 10.1016/j.csbj.2023.11.039 (PMC10697999; doi:10.1016/j.csbj.2023.11.039)
Supplement: Supplementary file 3 — Supplementary material [file mmc3.docx]

Supplementary material

| Query reaction | Sim_RF matches | Sim_2018 matches | Sim_RF  output and EC number | Sim_2018  output and EC number |
| --- | --- | --- | --- | --- |
| Incorrectly assigning isomerase reaction | | | | |
| MNXR163777*  MNXR145447*  1.14.14.154 | 0 | 1 | MNXR145451  5.4.99.7 | MNXR149263  1.3.1.70 |
| MNXR149263  1.3.1.70 | 0 | 1 | MNXR113311  5.4.99.52 | MNXR145449  1.14.13.70 |
| MNXR108351  1.14.11.31 | 0 | 1 | MNXR166220  5.3.99.6 | MNXR148251  1.1.1.218  1.1.1.247 |
| MNXR149811  1.14.19.20 | 0 | 2 | MNXR190810  5.3.3.5 | MNXR108813  1.1.1.170  1.1.1.270  1.14.13.72  1.14.18.9 |
| MNXR124172  2.2.1.1 | 0 | 3 | MNXR114630  5.3.1.34 | MNXR146501  2.2.1.2 |
| EC number discrepancies | | | | |
| MNXR97218  4.1.2.15, 2.5.1.54, 2.5.1.55 | 0 | 3 | MNXR122473  1.13.11.79 | MNXR125673  4.1.2.22 |
| MNXR112059*  1.14.14.32  MNXR102254*  1.14.99.9 | 2 | 3 | MNXR102258  1.14.13.54 | MNXR108152  1.14.14.1 |
| MNXR192629  2.5.1.78 | 3 | 4 | MNXR179128  2.5.1.9 | MNXR192628  2.5.1.78 |
| Underlying differences between the algorithms | | | | |
| MNXR107963  1.1.1.219 | 0 | 1 | MNXR141735  2.5.1.18 | MNXR110394  1.17.1.3 |
| MNXR100727  5.3.3.1, 1.1.1.145 | 3 | 4 | MNXR178243  1.1.1.51 | MNXR150710  2.5.1.18  5.3.3.1 |
| MNXR102547  5.4.2.1  5.4.2.11  5.4.2.12 | 3 | 4 | MNXR192018  5.4.2.2  5.4.2.5  5.4.2.8 | MNXR126095  5.4.2.1  5.4.2.11  5.4.2.12 |
| Similar reactions | | | | |
| MNXR188641  3.1.1.31 | 3 | 4 | MNXR169903  3.1.1.25 | MNXR192435  3.1.1.31 |

Supplementary Table 2 Instances where sim_2018 outperformed sim_RF. * indicate redundant reactions.
